# Spinal 5-HT_3_ receptors mediate descending facilitation and contribute to behavioral hypersensitivity via a reciprocal neuron-glial signaling cascade

**DOI:** 10.1186/1744-8069-10-35

**Published:** 2014-06-09

**Authors:** Wei Guo, Kan Miyoshi, Ronald Dubner, Ming Gu, Man Li, Jian Liu, Jiale Yang, Shiping Zou, Ke Ren, Koichi Noguchi, Feng Wei

**Affiliations:** 1 Department of Neural and Pain Sciences, Dental School; Program in Neuroscience, University of Maryland, 650 W. Baltimore St, Baltimore, Maryland 21201, USA; 2 Department of Anatomy and Neuroscience, Institute for Advanced Medical Sciences, Hyogo University, Hyogo 663-8501, Japan; 3 Department of Anesthesiology, Tongji Hospital, Tongji Medical College, Huazhong University of Science and Technology, Wuhan 430030, China; 4 Department of Anesthesiology, Jinling hospital, Nanjing University School of Medicine, Nanjing 210002, China

**Keywords:** 5-HT_3_ receptor, Glia, Proinflammatory cytokines, NMDA receptor, Pain

## Abstract

**Background:**

It has been recently recognized that the descending serotonin (5-HT) system from the rostral ventromedial medulla (RVM) in the brainstem and the 5-HT_3_ receptor subtype in the spinal dorsal horn are involved in enhanced descending pain facilitation after tissue and nerve injury. However, the mechanisms underlying the activation of the 5-HT_3_ receptor and its contribution to facilitation of pain remain unclear.

**Results:**

In the present study, activation of spinal 5-HT_3_ receptors by intrathecal injection of a selective 5-HT_3_ receptor agonist SR 57227 induced spinal glial hyperactivity, neuronal hyperexcitability and pain hypersensitivity in rats. We found that there was neuron-to-microglia signaling via the chemokine fractalkine, microglia to astrocyte signaling via cytokine IL-18, astrocyte to neuronal signaling by IL-1β, and enhanced activation of NMDA receptors in the spinal dorsal horn. Glial hyperactivation in spinal dorsal horn after hindpaw inflammation was also attenuated by molecular depletion of the descending 5-HT system by intra-RVM Tph-2 shRNA interference.

**Conclusions:**

These findings offer new insights into the cellular and molecular mechanisms at the spinal level responsible for descending 5-HT-mediated pain facilitation during the development of persistent pain after tissue and nerve injury. New pain therapies should focus on prime targets of descending facilitation-induced glial involvement, and in particular the blocking of intercellular signaling transduction between neurons and glia.

## Background

Recent studies indicate that behavioral hypersensitivity and neuronal hyperexcitability in the CNS in animal models of persistent pain are closely linked to long-lasting activation of descending modulatory circuits involving descending facilitation ([1-5] See [6-10] for reviews). It has been well established that the descending serotonin (5-HT) system from the rostral ventromedial medulla (RVM) of the brainstem is involved in the modulation of spinal nociceptive transmission [11-14]. Selective lesions of spinal 5-HT fibers [15] or molecular depletion of 5-HT in RVM neurons [16] have been reported to attenuate behavioral hypersensitivity following injury. These effects of the descending 5-HT system resulted from the activation of diverse 5-HT receptor subtypes found in the spinal dorsal horn [17-19]. 5-HT_3_ receptors, the only ligand-gated cation channel with excitatory functions in the 5-HT receptor family, are expressed in spinal dorsal horn neurons and the central terminals of primary afferent neurons [20,21]. Spinal 5-HT_3_ receptor-dependent descending pain facilitation has recently been implicated in the development of inflammatory and neuropathic pain [5,19,22-25]. However, the signaling cascade underlying the contribution of spinal 5-HT_3_ receptors to descending pain facilitation remains unclear.

Ample evidence suggests that glial cells in the spinal cord contribute to pain hypersensitivity after injury [26-30]. In addition to glutamate, spinal neurons and the central terminals of primary afferents release chemokines, such as fractalkine (CX3CL1), activating nearby glial cells [31,32]. Furthermore, hyperactivated glia amplify neuronal excitability and facilitate nociceptive transmission in spinal cord via release of pro-inflammatory cytokines (e.g. IL-1β and TNF-α) [33-35]. Increasing attention has been given to neuron-glia-neuron signaling as a driving force in the development and maintenance of persistent pain [26-30].

Utilizing a model of 5-HT_3_ receptor agonist-induced hyperalgesia, we tested the hypothesis that neuron-glial interactions involving chemokine/cytokine signaling molecules underlie mechanisms of pain hypersensitivity after spinal 5-HT_3_ receptor activation. Our findings provide evidence that a spinal neuron-glia-neuron signaling cascade including endogenous fractalkine, the cytokines IL-18 and IL-1β, and neuronal GluN (NMDA) receptor activation, contribute to 5-HT_3_ receptor-mediated hyperalgesia. Thus, spinal neuron-glial interactions underlying the development of hyperalgesia and allodynia not only depend on nociceptive drive from primary afferents after tissue and nerve injury [35,36], but also require maintenance of descending facilitation from RVM 5-HT-spinal 5-HT_3_ receptor systems.

## Results

### Activation of spinal 5-HT_3_ receptors induces hyperalgesia and allodynia

**Figure 1:**
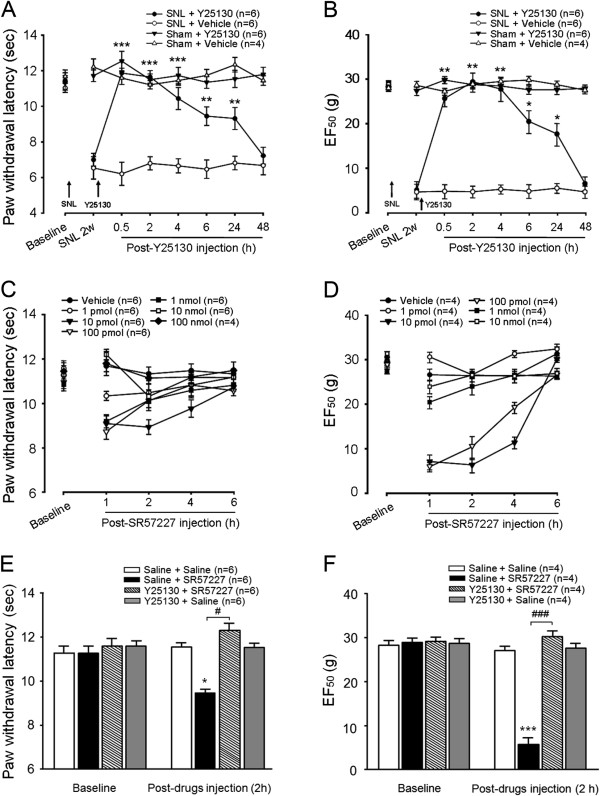
**Attenuation of nerve injury- or intrathecal 5-HT3 receptor agonist SR57227-induced behavioral hypersensitivity by intrathecal treatment of 5-HT3 receptor antagonist Y25130. A**-**B**: Nerve injury-induced thermal hyperalgesia **(A)** and mechanical allodynia **(B)** at 2 weeks after the left L5 spinal nerve ligation were significantly attenuated for 24 h after post-treatment of Y25130 (30 fmol, i.t.) compared with vehicle. ***, p < 0.001, **, p < 0.01, SNL + Y25130 vs. SNL + vehicle. n = 4–6 per group. **C**-**D**: SR57227 (10 pmol -1 nmol) induced significant decreases in thermal paw withdrawal latencies (PWLs) **(C)** and mechanical EF 50s **(D)** after intrathecal injection compared with vehicle saline (*, p < 0.05, n = 6 rats per group). Note that SR57227 at the 10 pmol dose produced a longer thermal hyperalgesia and mechanical allodynia lasting for 4 h; at a higher dose (10 nmol), SR57227 induced a transient hypoalgesia and at a lower dose (1 pmol) did not change thermal nociception **(C)**. **E**: At 2 h time point, SR57227-induced thermal hyperalgesia was completely blocked by pretreatment of 5-HT_3_ receptor antagonist Y25130 (30 fmol, i.t.) but not saline, each of which was injected 30 min before the injection of SR57227 (10 pmol, i.t.) **F**: SR57227-induced mechanical hypersensitivity was totally reversed by pretreatment of Y25130 (30 fmol, i.t.) at 2 h after injection. *, p < 0.05, ***, p < 0.001, vs. saline + saline; ^#^, p < 0.05, ^###^, p < 0.001, vs. saline + SR57227; n = 4–6 per group).

Our previous study demonstrated that descending 5-HT-dependent pain facilitation contributes to behavioral hyperalgesia and allodynia after peripheral inflammation and nerve injury [16,37,25]. Recently, we also found that the spinal 5-HT_3_ receptor mediated the development of pain hypersensitivity after inflammation induced by hindpaw injection of complete Freund’s adjuvant (CFA) [24] and maintained persistent pain states after trigeminal nerve injury [25,37]. To further confirm an involvement of the spinal 5-HT_3_ receptor in persistent pain, we examined the effect of the blockade of spinal 5-HT_3_ receptor function on the maintenance of pain hypersensitivity in the rat spinal nerve ligation (SNL) model. Intrathecal injection (i.t.) of the selective 5-HT_3_ receptor antagonist Y25130 (30 fmol) alone did not produce an effect on baseline of thermal and mechanical sensitivity in sham animals (Figure 1A and B), shown by withdrawal latencies (PWLs) to noxious heat (Figure 1A) and withdrawal threshold (EF_50_) to mechanical stimulation (Figure 1B), suggesting an absence of tonic activation of spinal 5-HT_3_ receptors in the rats without injury. However, this dose of Y25130 significantly and reversibly attenuated SNL-induced thermal hyperalgesia and mechanical allodynia at least for 24 h when compared with the response in vehicle-treated rats (Figure 1A and B), indicating that spinal 5-HT_3_ receptors mediate descending pain facilitation during the development of persistent pain. To mimic the direct effect of activating the spinal 5-HT_3_ receptors on the behavioral pain response, we also intrathecally injected the selective 5-HT_3_ receptor agonist SR57227 and measured its influence on thermal and mechanical sensitivity of the hindpaw of the rat (Figure 1C and D). SR57227 induced significant thermal hyperalgesia as compared to vehicle (p < 0.05, n = 6 rats per group), in a range of doses from 10 pmol to 1 nmol (Figure 1C). The hypersensitive effect was maximal at the dose of 10 pmol, peaking at 1–2 h, lasting at least for 4 h. A lower dose (1 pmol) was without effect on PWLs. Conversely, a higher dose (10 nmol) of SR57227 produced a transient increase of thermal thresholds to noxious heat. Meanwhile, intrathecal SR57227 significantly resulted in mechanical hypersensitivity at 1–4 h after injection, in a similar range of doses (Figure 1D). To verify whether SR57227-induced behavioral hypersensitivity was mediated by 5-HT_3_ receptors, Y25130 (30 fmol), a selective 5-HT_3_R receptor antagonist, was injected intrathecally at 30 min before SR 57227 (10 pmol). The pretreatment of Y25130 completely blocked the thermal hypersensitive effect of SR 57227 at 1–4 h (Figure 1E; p < 0.05, n = 6) and mechanical allodynia at 2 h compared to pretreatment with vehicle (Figure 1F; p < 0.001, n = 4). This dose-exploration study in rats indicates that activation of the spinal 5-HT_3_ receptors induces long-lasting hyperalgesia and allodynia, supporting recent findings that spinal 5-HT_3_ receptor activation is involved in the development of descending pain facilitation.

### Selective activation of spinal 5-HT_3_ receptors induce hyperactivity of microglia and astrocytes

**Figure 2:**
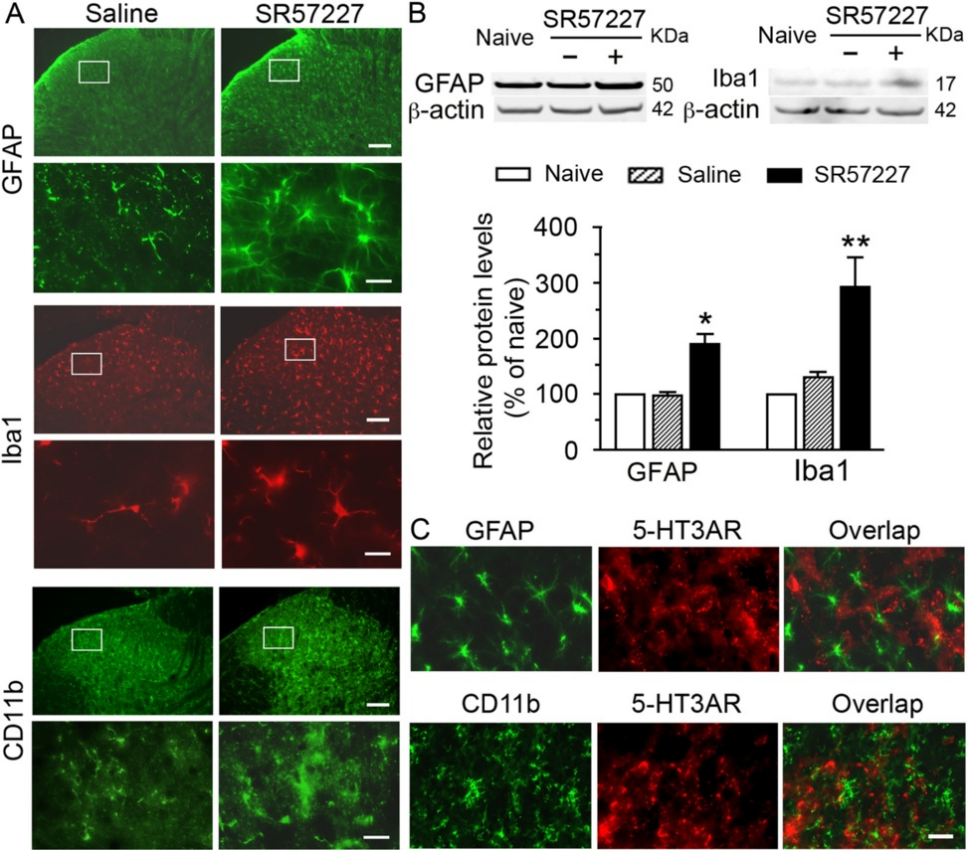
**Selective activation of 5-HT**_**3 **_**A receptors induces increase of both GFAP and Iba1 expression in the spinal dorsal horn but not in glial cells. A**. Immunoreactivity of GFAP, a marker of astrocytes, and Iba1 or CD11b, markers of microglia. Upper panels are of the spinal dorsal horn of rats at 2 h after receiving 10 pmol SR 57227 or vehicle. An increased expression of GFAP, Iba1 or CD11b was observed in an enlarged superficial dorsal horn (lower panels) corresponding to the small rectangle area in upper panels when compared to the vehicle-treated rats. **B**. Western blots at the expected molecular weights illustrate significant increases in the levels of GFAP and Iba1 in the spinal dorsal horn of rats at 2 h after intrathecal injection of SR57227 (10 pmol) compared with rats receiving saline. Representative blots are shown above and relative protein levels (% of naive) are shown in the bottom histograms (*, p < 0.05, **, p < 0.01, vs. saline group, n = 3 per group). **C**. Double immunostaining shows that SR57227 (10 pmol, i.t.)-induced GFAP or CD11b expression in the dorsal horn astrocytes or microglial cells was not colocalized with 5-HT_3_A receptor immunoreactivity. Scale bar = 25 μm.

What are possible mechanisms underlying spinal 5-HT_3_ receptor activation-induced hyperalgesia and allodynia? In the spinal cord glial activity is critical for the induction and maintenance of hyperalgesia after tissue and nerve injury. Thus, we hypothesized that 5-HT_3_ receptor-induced hyperalgesia and allodynia involved changes in the activity of spinal glial cells. Biochemical markers for microglia (Iba1 or CD11b) and astrocytes (GFAP) were used to determine the location of glial expression using immunohistochemistry (Figure 2A) and to assess the changes in expression using Western blotting (Figure 2B) after spinal 5-HT_3_ receptor activation. The microglia and astrocytes exhibited hypertrophy with thicker processes and larger and densely stained cell bodies in spinal dorsal horn at L5 2 h after intrathecal injection of SR 57227 (10 pmol) in comparison with vehicle treatment (Figure 2A). Consistently, a significantly enhanced expression pattern of both GFAP and Iba1 was identified by Western blot analysis in the lumbar spinal dorsal horn at 2 h after application of SR 57227 (Figure 2B). However, we did not find significant changes in CD11b expression in the dorsal horn tissue after intrathecal injection of SR 57227 (data not shown). These results suggest that there is functional hyperactivity or reactivation of microglia and astrocytes in the spinal dorsal horn after direct activation of local 5-HT_3_ receptors.

### Selective expression of 5-HT_3_ receptors in neurons but not glia

**Figure 3:**
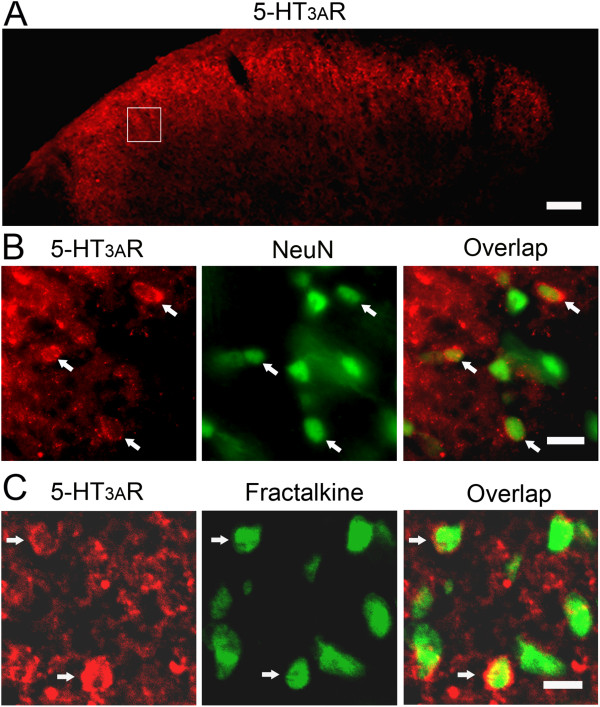
**5-HT**_**3**_**A receptor expression in spinal dorsal horn neurons. A**. Distribution of 5-HT_3_A receptor expression in the spinal dorsal horn under low magnification. Intense expression of 5-HT_3_A receptor immunoreactivity was mainly observed in the superficial layers of the spinal dorsal horn. **B**. Double immunostaining showing 5-HT_3_A receptor profiles also labeled with NeuN, a neuronal marker in the dorsal horn cells (arrows), from the inset of **A**. **C**. Colocalization of immunoreactivity of 5-HT_3_A receptor and fractalkine in the spinal dorsal horn cells. Arrows indicate examples of double-labeled profiles. Scale bar = 100 μm **(A)** and 25 μm **(B and C)**.

Since SR57227 induced astrocytic and microglial hyperactivity, we wondered whether this compound directly acted on glial cells to produce its effects. Although the existence of the 5-HT_3_ receptor has been reported in neuronal soma and terminals in the dorsal horn [20,21,25], it is not known whether the 5-HT_3_ receptor is also expressed in spinal glial cells. Therefore, we examined the distribution of the 5-HT_3_A receptor in the spinal cord and its possible expression in glial cells. As shown in Figure 3A, intense immunoreactivity of the 5-HT_3_A receptor was observed in the superficial layers of the spinal dorsal horn. In addition, weak to moderate expression of 5-HT_3_ receptors was scattered throughout the spinal cord. Western blot analysis showed no significant differences in the level of 5-HT_3_ receptor among groups of naïve rats and rats in which SR 57227 or saline was intrathecally injected (n = 3 per group, data not shown). Double immunolabeling with 5-HT_3_A receptor and GFAP or CD11b showed no expression of the 5-HT_3_ receptor in astrocytes and microglia including glial soma and processes (Figure 2C). Similarly, labeling for the 5-HT_3_ receptor was not seen in both hyperactive astrocytic and microglial elements in the spinal dorsal horn following administration of SR 57227 (data not shown). In contrast, 5-HT_3_A receptors were distributed throughout neuronal soma and many neurites in the spinal dorsal horn, typically as small clusters associated with the cell membrane of the neurons labeled with NeuN (Figure 3B). Consistent with previous reports [20,21], these data confirm that 5-HT_3_ receptors are primarily expressed in some neuronal soma and terminals but not glial cells in the spinal dorsal horn.

### 5-HT_3_ receptor-labeled neurons express fractalkine in the dorsal horn

**Figure 4:**
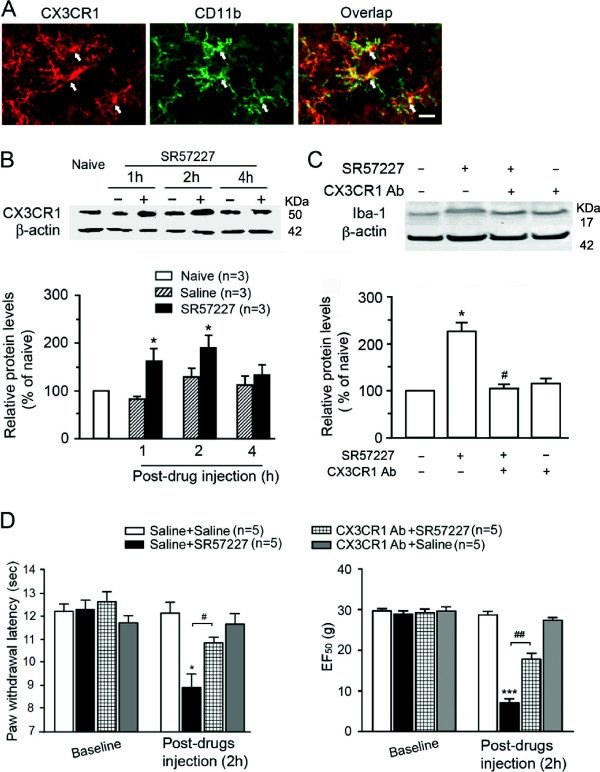
**Attenuation of SR57227-induced upregulation of fractalkine receptor CX3CR1 in spinal microglia and behavioral hypersensitivity to a neutralizing antibody against CX3CR1. A**. CX3CR1 immunoreactivity exists in all dorsal horn microglial cells (arrows) expressing CD11b at 2 h after intrathecal SR57227 (10 pmol). Scale bar = 25 μm. **B**. Western blot analysis showing a significant upregulation in the levels of CX3CR1 in the spinal dorsal horn tissue in rats at 1 and 2 h after intrathecal injection of SR57227 (10 pmol) but not vehicle saline (*, p < 0.05, vs. saline, n = 3 per group). **C**. SR57227-induced increases in the protein levels of Iba1 in the spinal dorsal horn tissue were significantly blocked by a neutralizing antibody against CX3CR1 (CX3CR1 Ab, 20 μg) at 2 h after co-administration (*, p < 0.05, vs. saline + saline; ^#^, p < 0.05, CX3CR1 Ab + SR57227 vs. saline + SR57227; n = 3 per group). **D**. Thermal (left) and mechanical (right) hypersensitivity induced by SR57227 (10 pmol, i.t.) was partially eliminated by pretreatment with a CX3CR1 neutralizing antibody (Ab) (20 μg, i.t.) 1 d before and concurrently with SR57227 at 2 h after injection of SR 57227. *, p < 0.05, vs. saline + saline; #, p < 0.05, CX3CR1 Ab + SR57227 vs. saline + SR57227, n = 5 per group).

In view of the absence of 5-HT_3_A receptors in glial cells of the spinal dorsal horn, we reasoned that 5-HT_3_ receptors-expressing neurons or terminals may mediate SR57227-induced glial hyperactivity by releasing neuroactive substances. The chemokine, fractalkine (CX3CL1) has been found in sensory afferents and intrinsic spinal cord neurons [38] whereas its receptor, CX3CR1, is expressed predominantly in microglia [39-41] and may act as a specific neuron-to-glia signal in the spinal cord [32]. Therefore, to identify the possible participation of fractalkine as a signaling molecule between neuron and glia in SR 57227-induced behavioral hypersensitivity and glial hyperactivity, we investigated the expression of fractalkine in 5-HT_3_A receptor-labeled neurons in the spinal dorsal horn. Immunoreactivity of fractalkine was observed in numerous dorsal horn neurons (Figure 3C) and terminals (data not shown). Double staining indicated that all 5-HT_3_A receptor-labeled neuronal soma in the dorsal horn strongly expressed fractalkine (Figure 3C). Consistent with previous observations [39,40], we identified the colocalization of CX3CR1 in microglia in the spinal dorsal horn by double labeling for CX3CR1 and CD11b (Figure 4A). These data suggest that fractalkine may directly mediate signaling from 5-HT_3_ receptor-expressing neurons to microglia.

### Up-regulated CX3CR1 and activated microglia contribute to SR57227-induced hyperalgesia/allodynia and glial hyperactivity

To assess the role of CX3CR1 in downstream events subsequent to 5-HT_3_ receptor activation, we measured the change of tissue CX3CR1 expression in the spinal dorsal horn after intrathecal injection of SR57227. Western blot analysis showed a transient up-regulation of CX3CR1 level after application of SR 57227 (10 pmol, i.t.) compared with saline (p < 0.05, n = 3 for each group) (Figure 4B). Furthermore, to identify whether CX3CR1 activation mediates SR57227-induced increase of expression of Iba1 protein, we examined the effect of a neutralizing antibody for CX3CR1 on SR57227-induced microglial hyperactivity. This neutralizing antibody (CX3CR1 Ab, 20 μg), intrathecally injected at 1d before and concurrently with SR57227 (10 pmol), significantly attenuated the enhancement of spinal Iba1 expression at 2 h induced by application of SR57227 (Figure 4C) (p < 0.05, n = 3). Next, we also examined the effect of blockade of CX3CR1 on SR57227-induced behavioral hypersensitivity. Consistent with a finding that CX3CR1 KO mice showed an attenuation of mechanical and thermal hypersensitivity after nerve injury when compared with CX3CR1 WT mice [42], the pretreatment with CX3CR1 Ab (20 μg, i.t.) significantly attenuated the thermal hypersensitivity (Figure 4D left panel) (p < 0.05, n = 5 for each groups) and the mechanical allodynia at 2 h (Figure 4D right panel) (p < 0.001, n = 5 for each groups) after application of SR57227. Injection of the antibody alone did not affect PWLs and EF50 (Figure 4D). However, this is in contrast to the finding reported by Staniland and colleagues that CX3CR1 KO mice displayed only a loss of thermal hypersensitivity in a model of inflammation induced by intraplantar injection of zymosan [42], suggesting that the fractalkine-CX3CR1 signaling cascade can be differentially affected depending on the pathological pain model and the stimulus modality. All of the above findings implicate the fractalkine-CX3CR1 signaling cascade in neuron-microglial interaction during glial hyperactivity and behavioral hypersensitivity after neuronal 5-HT_3_ receptor activation at the spinal level.

**Figure 5:**
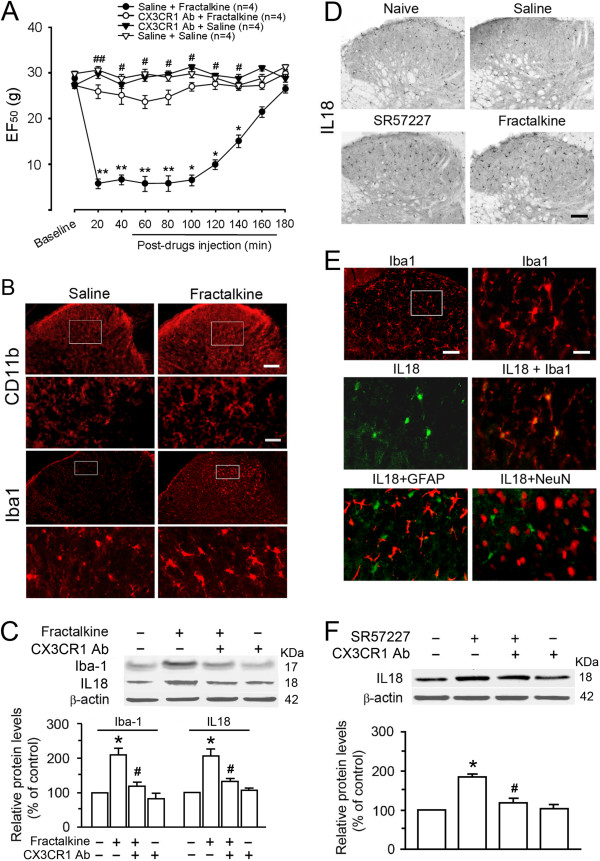
**Fractalkine-induced hypersensitivity and up-regulation of IL-18 are mediated by spinal microglia. A**. Fractalkine (40 ng, i.t.) induced behavioral hyperalgesia (p<0.001, one-way ANOVA, n = 4 for each group; *, p<0.05, **, p<0.01, vs. baseline), which was significantly attenuated by pretreatment with a neutralizing antibody against CX3CR1 (CX3CR1 Ab, 20 μg, i.t.) (^#^, p<0.05, ^##^, p<0.01 vs. saline + fractalkine). **B**. Fractalkine (40 ng) increased CD11b or Iba1 expression (lower panels); lower panels are from the insets of the spinal dorsal horn (upper panels, respectively) at 1 h after intrathecal injection when compared with vehicle treatment. Scale bar=100 μm (upper panels) and 25 μm (lower panels). **C**. The expression of Iba1 and IL18 in the spinal dorsal horn was increased at 2 h after injection of fractalkine (40 ng, i.t.) when compared with saline group (*, p<0.05, n=3 per group); such upregulation was significantly attenuated by pretreatment of CX3CR1 Ab (20 μg, i.t.) (^#^, p<0.05, n=3). **D**. IL18 in the dorsal horn was upregulated 2 h after i.t. SR57227 (10 pmol) or fractalkine (40 ng) but not vehicle when compared with that in naïve rat (n=3-4 per group). Scale bar=100 μm. **E**. Increased expression of Iba1 but not GFAP in the dorsal horn glial cells was colocalized with IL-18-IR at 2 h after fractalkine treatment (40 ng, i.t. n=3-4). There was no coexpression of IL-18 and NeuN. The right upper panel is an enlarged area from the inset from the dorsal horn in the left upper panel. Scale bar=100 μm (the left upper panel) and 25μm (the right upper, middle and lower panels). **F**. IL-18 was increased in the dorsal horn at 2 h after i.t. SR57227 (10 pmol, n=3) (*, p<0.05) compared with that with saline (i.t., n=3). CX3CR1 Ab (20 μg, i.t., n=3) prevented the effects of SR57227 on IL-18 expression (^#^, p<0.05).

We then tested whether the effect induced by endogenous fractalkine release from 5-HT_3_ receptor-activated neurons was mimicked by application of exogenous fractalkine. Intrathecal delivery of fractalkine (40 ng/10 μl), but not vehicle, produced a significant mechanical allodynia that developed within 20 min and lasted for up to 3 h (p < 0.05 or 0.01 vs. saline + saline, n = 4 per group) (Figure 5A) and a similar pattern of thermal hyperalgesia peaking at 40–60 min after injection (data not shown). In addition, both immunostaining and Western blotting analysis showed an enhanced hyperactivity of microglia at 1 h after the delivery of fractalkine, along with increased expression of Iba1 or CD11b (Figure 5B and C) in the spinal dorsal horn. Moreover, the hyperalgesic effect of fractalkine or the induced enhancement of spinal Iba1 expression was significantly attenuated by pretreatment with CX3CR1 Ab at 20 to 140 min (p < 0.05 vs. saline + fractalkine) (Figure 5A) or 1 h (Figure 5C) after injection, respectively. Thus, the functional effects of blockade of CX3CR1 activation on fractalkine-induced increase of Iba1 and behavioral hypersensitivity further support our hypothesis that endogenous fractalkine released from 5-HT_3_ receptor-containing neurons or terminals results in microglial activation by acting on its receptor CX3CR1 mainly expressed on microglia in the dorsal horn.

### Up-regulated IL-18 in microglia and IL-18 receptor in astrocytes mediate microglia-astrocytic interaction during SR57227-induced hyperalgesia and allodynia

Hyperactive microglia are known to synthesize and secrete many glioactive substances such as proinflammatory cytokines involved in microglia-astrocytic interaction, the modulation of neuronal activity and the enhancement of hypersensitivity to noxious input. Thus, we further identified downstream effects of spinal microglial activation after intrathecal administration of SR57227 or fractalkine. Although many chemical mediators including proinflammatory cytokines have been found to be involved in microglia-dependent signaling cascades, we speculated that IL-18 may contribute to the downstream effects because of its unique expression in microglia and its receptor primarily found in astrocytes in the spinal dorsal horn as well as its crucial role in glial mechanisms underlying the development and maintenance of mechanical allodynia [43]. Thus, we determined whether the IL-18/IL-18 receptor signaling pathway in spinal microglia-astrocyte interaction contributed to SR 57227- or fractalkine-induced pain hypersensitivity. Western blot analysis demonstrated that selective activation of CX3CR1 by intrathecal fractalkine resulted in significant increase of IL-18 expression in the spinal dorsal horn (Figure 5C) when compared with vehicle treatment (p < 0.05, n = 3 for each group), which was also suppressed by pretreatment with CX3CR1 Ab (p < 0.05, vs. saline + fractalkine) (Figure 5C). Consistently, higher intensity of IL-18 immunoreactivity was visualized in the dorsal horn cells at 2 h after intrathecal fractalkine but not vehicle, compared to that in the naïve condition (Figure 5D). Double immunostaining further confirmed that IL-18 was predominantly expressed in microglia labeled by Iba1 immunoreactivity in the dorsal horn (Figure 5E), consistent with previous observations [43]. There was little or no colocalization of IL-18 with GFAP or NeuN (Figure 5E). Moreover, we evaluated the changes of IL-18 during microglial hyperactivity after intrathecal injection of SR57227. Similar to the effect of fractalkine, activation of spinal 5-HT_3_ receptors induced significant increase of IL-18 immunoreactive intensities shown by immunostaining (Figure 5D) or IL-18 protein levels measured by Western blots (Figure 5F) in the dorsal horn. Compared to that in vehicle group, SR57227-enhanced IL-18 expression was significantly prevented by pretreatment with intrathecal CX3CR1 Ab (Figure 5F) or blocked by Y25130 (p < 0.05, 136.7 ± 3.5% in Y25130 + SR57227 vs. 187.2 ± 6.7% in saline + SR57227). Treatment of Y25130 alone did not change baseline expression of IL18 in spinal dorsal horn (96.7 ± 4.5%, p > 0.05, vs. vehicle group) (n = 3 for each group).

**Figure 6:**
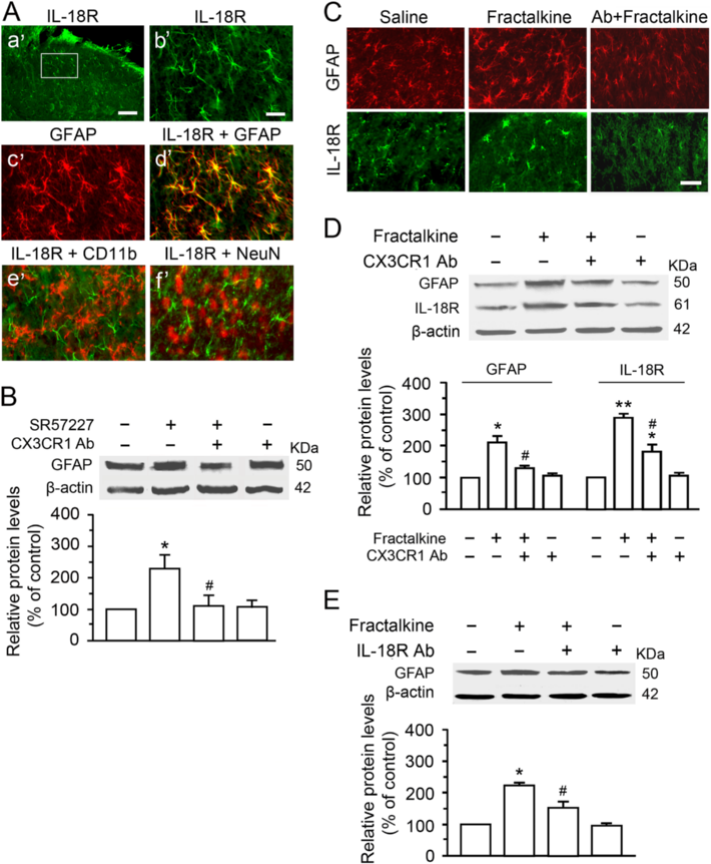
**Astrocytic hyperactivity and up-regulation of IL-18R in spinal astrocytes induced by SR57227 and fractalkine. A**. IL-18R expression in the dorsal horn (a’ and b’) and its colocalization (d’) with GFAP (c’) but not with CD11b (e’) or NeuN (f’), suggesting that IL-18R is only expressed in spinal astrocytes of normal rats. Scale bar=100 μm (a’) and 25 μm (b’-f’). **B**. SR57227 (10 pmol, i.t.) induced a significant enhancement of GFAP expression in the dorsal horn at 1 h (*, p<0.05, vs. vehicle, n=3 per group), which was suppressed by pretreatment with CX3CR1 Ab (20 μg, n=3) (^#^, p<0.05 vs. saline + SR57227). **C**. Fractalkine (40 ng, i.t., n=4)-induced increases of both GFAP (upper panels) and IL-18R (lower panels) expression were significantly attenuated by pretreatment with CX3CR1 Ab (Ab, 20 μg, i.t., n=3) at 1 h after injection of fractalkine. Scale bar=25 μm. **D** The levels of IL-18R and GFAP are significantly increased in the dorsal horn at 1 h after fractalkine treatment (40 ng, i.t., n=3) compared with that treated by saline (*, p<0.05, or **, p<0.01, n=3 for each group); such upregulation for GFAP was significantly reduced by pretreatment with CX3CR1 Ab (20 μg, ^#^, p<0.05, vs. saline + Fractalkine, n=3 per group); fractalkine-induced increase of IL-18R expression was also attenuated by CX3CR1 Ab (^#^, p<0.05, n=3) but did not return to basal expression found in the saline + saline treated group (*, p<0.05). **E**. Fractalkine-induced increase of GFAP expression in the dorsal horn was partially suppressed by pretreatment with IL-18 Ab (20 μg, i.t.) (*, p<0.05, vs. saline + saline; ^#^, p<0.05, vs. saline + fractalkine; n=3 for each group). These data suggest that SR57227 or fractalkine-induced up-regulation of GFAP is mediated by activation of CX3CR1 expression in spinal microglia and of IL-18R in astrocytes.

In contrast to IL18 expression in microglia, the IL-18 receptor (IL-18R) was present in GFAP labeled astrocytes but not in microglia and neurons (Figure 6A). Thus, spinal IL-18R expression during glial hyperactivity after intrathecal injection of SR57227 or fractalkine was examined. Immunostaining showed an increase of GFAP expression accompanied by enhanced intensity of IL-18R immunoreactivity in the dorsal horn cells after intrathecal injection of fractalkine (40 ng) when compared to that treated by vehicle (Figure 6C). Pretreatment with CX3CR1 Ab reduced fractalkine-induced increases of spinal GFAP and IL-18R expression (Figure 6C). Quantification of this effect was obtained with Western blotting for fractalkine-induced upregulation of astrocytic IL-18R in the dorsal horn, which were significantly blocked by pretreatment with CX3CR1 Ab (Figure 6D). In order to verify whether a hyperactivity of spinal astrocytes was downstream of spinal microglial activation, we also analyzed the effects of functional blockade of these cytokine receptors primarily expressed on microglia or astrocytes on the enhanced expression of GFAP induced by SR57227 and fractalkine. We found that SR57227-induced elevation of GFAP expression was significantly attenuated after blocking the fractalkine-mediated signal transduction cascade with anti-CX3CR1 antibody (p < 0.05, Figure 6B). Also, fractalkine-induced GFAP increase was significantly attenuated after functional blockades of either CX3CR1 expression in microglia (Figure 6D) or IL-18 receptors expressing in astrocytes (Figure 6E). These data suggest an increase in the functional interaction between microglial and astrocytic hyperactivity via a key IL-18/IL-18R signaling pathway during the development of hyperalgesia induced by 5-HT_3_ receptor activation and exogenous fractalkine.

### Up-regulated IL-1β in astrocytes mediates SR57227-induced pain behavior through phosphorylation of neuronal GluNRs (NMDARs)

**Figure 7:**
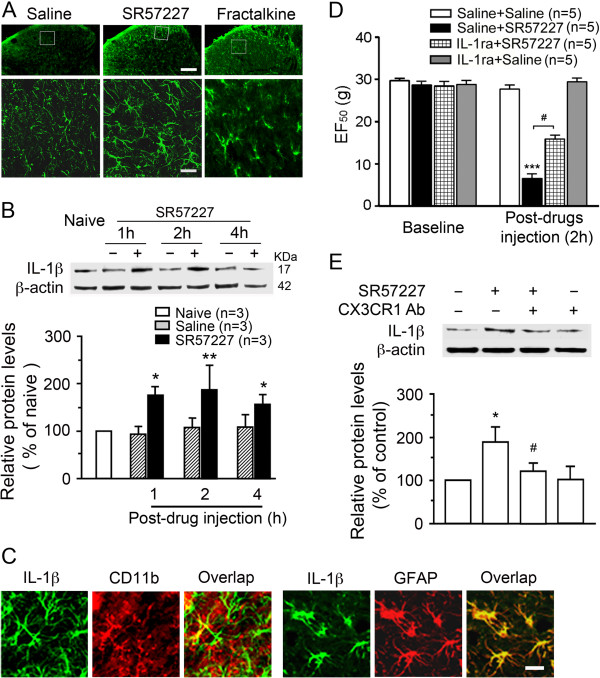
**Upregulation of IL-1β in the dorsal horn astrocytes and its involvement in pain behavior after intrathecal injection of SR57227. A**. SR 57227 (10 pmol, i.t., n = 4) or fractalkine (40 ng, i.t., n = 4) produced an increased expression of IL-1β (lower panels) in the area from the inset in the spinal dorsal horn (upper panels) compared to saline at 2 h or 1 h after injection, respectively. Scale bar = 100 μm (upper panel) and 25 μm (lower panel). **B**. Western blot analysis showed increases in the levels of IL-1β in the spinal dorsal horn tissue of rats treated with SR 57227 (10 ng, i.t., *, p < 0.05 at 1 and 4 h, **, p < 0.01 at 2 h, vs. saline, n = 3 per group). **C**. Dense colocalization of IL-1β and GFAP (lower panels) but not CD11b in glial cells (upper panels) in the spinal dorsal horn in rats treated with SR 57227 (10 pmol, i.t., n = 3) at 2 h after injection, suggesting that IL-1β was predominantly expressed in spinal astrocytes. Scale bar = 25 μm. **D**. SR 57227 (10 pmol, i.t.)-induced mechanical hypersensitivity was attenuated at 2 h after the injection by the antagonist of IL-1 receptor, IL1-ra (10 μg, i.t.) 1d before and concurrently with SR 57227 (***, p < 0.001, saline + SR 57227 vs. saline + saline; ^#^ p < 0.05, IL1-ra + SR 57227 vs. saline + SR 57227) (n = 5 per group). **E**. SR 57227 (10 ng, i.t.) significantly induced up-regulation of IL-1β at 2 h after injection (*, p < 0.05, vs. saline + saline), which was attenuated by pretreatment with CX3CR1 Ab (20 μg, i.t.) (^#^, p < 0.05, vs. saline + SR57227) (n = 3 per group).

Hyperactivated microglia and astrocytes in the spinal dorsal horn and the brain stem trigeminal transition zone are known to secrete prototypic inflammatory cytokines such as IL-1β and are involved in central sensitization and behavioral pain hypersensitivity [44-47]. To test whether SR57227-induced hyperalgesia and allodynia involve IL-1β, we examined the effect of 5-HT_3_ receptor activation on spinal IL-1β expression and found that spinal delivery of SR57227 (10 pmol) but not vehicle induced a significant increase in IL-1β expression in dorsal horn cells (Figure 7A) or in the dorsal horn tissues (Figure 7B, p < 0.05 at 1 and 4 h, p < 0.01 at 2 h, vs. saline groups, n = 3 per group). A similar effect of exogenous fractalkine (40 ng) on spinal IL-1β expression was observed (Figure 7A). Next, we showed with double labeling that IL-1β was mainly expressed in hyperactive astrocytes immunoreactive for GFAP but rarely in microglia immunoreactive for CD11b in the spinal dorsal horn after injection of SR57227 (Figure 7C). We then examined whether IL-1β contributed to behavioral hypersensitivity induced by SR57227. IL-1ra (100 μg/10 μl), an IL-1 receptor antagonist, was injected intrathecally 1d before and concurrently with SR 57227. The SR57227-induced mechanical allodynia was significantly attenuated by pretreatment with IL-1ra at 2 h measured (Figure 7D). Thermal hyperalgesia induced by spinal 5-HT_3_ receptor activation was also reversed by intrathecal IL-1ra for 4 h (data not shown, p < 0.01 at 1 h or p < 0.05 at 2–4 h, n = 5 for each group). To demonstrate a one-way intercellular communication involving up-regulation of IL-1β evoked by spinal microglial and astrocytic activation, we blocked microglial receptor CX3CR1 or astrocytic IL-18 receptors and evaluated their effects on the increased expression of spinal IL-1β after intrathecal SR57227 or fractalkine. Western blotting showed that SR57227 induced enhancement of IL-1β expression in the dorsal horn tissue at 2 h after injection, when compared with vehicle treatment, and was significantly reduced with pretreatment with the CX3CR1 neutralizing antibody (p < 0.05, n = 3 for each group, Figure 7E). Meanwhile, fractalkine-induced increase of IL-1β level in the spinal dorsal horn was partially blocked by pretreatment with IL-18R Ab (p < 0.05, 128.2 ± 9.8% in IL-18R Ab + Fractalkine vs. 223.7 ± 7.6% in saline + Fractalkine, n = 3 for each group). Treatment of IL-18R Ab alone did not effect on basal IL-1β expression in spinal dorsal horn (96.7 ± 4.5%, p > 0.05, vs. vehicle group, n = 3 for each group). Thus, these data confirm that the enhanced expression of IL-1β in spinal astrocytes was induced by hyperactive microglia and activated IL-18 receptors in astrocytes in the dorsal horn.

**Figure 8:**
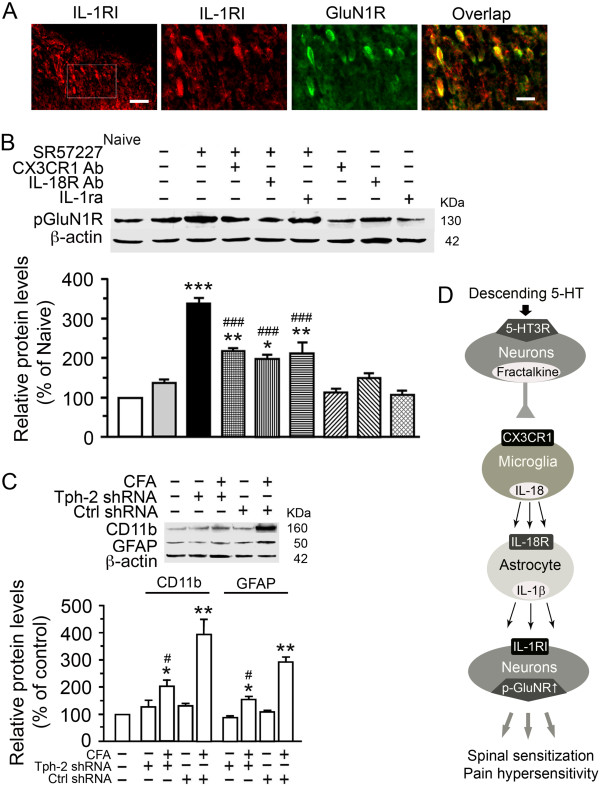
**Upregulation of NMDAR phosphorylation in dorsal horn after activation of 5-HT**_**3**_**Rs and descending 5-HT-dependent spinal glial hyperactivity after inflammation. A**. IL-1RI was colocalized with GluN1R in dorsal horn neurons. Enlarged regions (the three right panels) correspond to the rectangle area in the left panel. Scale bar=50 μm (the left panel) and 25 μm (the three right panels). **B**. p-GluN1R was increased in the dorsal horn at 2 h after i.t. SR57227 (10 pmol, i.t.; ***, p<0.001, n=3), which was significantly attenuated by pretreatment with CX3CR1 Ab (20 μg, i.t., n=3), IL18R Ab (20 μg, i.t., n=3) or IL-1ra (10 μg, i.t., n=3) (^####^, p<0.001 vs. saline + SR 57227; but *, p<0.05 or **, p<0.01, vs. saline + saline), respectively. **C**. CD11b and GFAP were increased in the dorsal horn after hindpaw injection of CFA in rats treated with control or Tph-2 shRNA in the RVM (*, <0.05 or **, p<0.01, vs. saline group), however, intra-RVM Tph-2 shRNA reduced CFA-produced increases of spinal CD11b and GFAP expression, in comparison with control Tph-2 shRNA (^#^, p<0.05) (n=3 for each group). Treatment with Tph-2 shRNA alone in the RVM didn’t affect basal expression of spinal CD11b and GFAP when compared to control shRNA. **D**. Proposed signal pathways involved in pain hypersensitivity after 5-HT_3_ receptor activation in the spinal cord. Some excitatory spinal neurons and primary afferent terminals expressing 5-HT_3_ receptors are activated by the 5-HT3R agonist. Fractalkine is released from the 5-HT3R-containing neurons or sensory afferents, and then acts on its receptor, CX3CR1 that is mainly expressed in microglia. Hyperactivity of microglia consequently evokes astrocytes through an IL18/IL18R signaling and induces astrocytic IL-1β release. The released IL-1β contributes to neuronal hyperexcitability and behavioral hypersensitivity via IL-1β receptors (IL-1RI) and enhancement of pGluN1R on spinal dorsal horn neurons.

It has been shown that glutamate receptor subunit GluN (NMDA) receptors (GluNRs) are widely expressed in rat dorsal horn neurons and are upregulated and phosphorylated in the dorsal horn by locally released proinflammatory cytokines after injury, contributing to pain hypersensitivity [35,47-49]. To study mechanism of glial-neuronal interactions, we examined whether IL-1RI, the receptor for IL-1β, was distributed in dorsal horn neurons containing GluNRs. Double labeling showed that IL1RI colocalized with the GluN1R subunit, a principal component of GluNRs in rat dorsal horn neurons (Figure 8A). To test whether spinal 5-HT_3_ receptor activation also induced GluNRs activation, the phosphorylation levels of GluN1R (pGluN1R), a functional marker of neuronal excitability in the CNS, were measured in spinal dorsal horn tissues. As shown in Figure 8B, the expression of pGluN1R ser896 in the spinal dorsal horn was robustly increased by threefold at 2 h after intrathecal injection of SR57227 (10 pmol) when compared with sham group (p < 0.001, n = 3 each group). Moreover, SR57227-induced increase of the pGluN1R expression was significantly attenuated by pretreatment with the neutralizing antibodies for CX3CR1, IL-18R or IL1RI (p < 0.001, Figure 8B), Meanwhile, these pretreatments alone had no effect on baseline expression of pGluN1 in the spinal dorsal horn (Figure 8B). These findings confirm that the increase of pGluN1 observed above was mediated mainly by fractalkine to CX3CR1, IL-18 to IL-18R and IL-1β to IL1RI signaling pathways, suggesting that spinal 5-HT_3_ receptor mediated hyperalgesia and allodynia in rat primarily depends on a neuron-microglia-astrocyte-neuronal signal cascade.

### Descending 5-HT and spinal 5-HT_3_ receptors involved in dorsal horn glial hyperactivity underlying descending pain facilitation and inflammatory pain

Recently, it has been well recognized that microglial and astrocytic hyperactivity in the dorsal horn play a critical role in the development of inflammatory pain after tissue injury [27-30] for reviews]. We previously found that the RVM-spinal 5-HT system is also implicated in descending pain facilitation involving in central mechanisms of persistent pain after peripheral inflammation [16] or nerve injury [25,37]. To confirm the effect of the descending 5-HT system on glial hyperactivity to peripheral inflammation in rats with persistent pain, we examined changes of spinal glial markers in a model of inflammatory pain induced by intraplantar injection of CFA from 3 d after molecular depletion of intra-RVM 5-HT system manipulated by local gene transfer of Tph-2 shRNA as shown previously [16]. In the control shRNA-treated rats, Western blot showed that there were robust increases of CD11b and GFAP expression in the spinal dorsal horn at 1 d after unilateral intraplantar CFA injection, compared with that in the saline group (p < 0.01, n = 3 for each group, Figure 8C). However, Tph-2 shRNA-treated animals exhibited significant attenuation of CD11b and GFAP expression after CFA injection as compared to rats treated with control shRNA (p < 0.05, n = 3 for each group, Figure 8C), suggesting that active 5-HT-dependent descending pain facilitation contributes to the maintenance of spinal glial hyperactivity underlying the development of inflammatory pain after injury. Thus, the spinal glial changes appear to be involved in descending facilitation underlying the maintenance of persistent pain via descending 5-HT release and spinal 5-HT_3_ receptor activation after injury.

## Discussion

Our findings demonstrate that a neuron-glia-cytokine-neuronal signaling cascade is involved in the mechanisms underlying spinal 5-HT_3_ receptor-mediated hyperalgesia. The development of persistent pain after inflammation and nerve injury appears to be dependent, in part, upon 5-HT pathways originating from the rostral ventromedial medulla leading to activation of 5-HT_3_ receptors at the spinal level [5,16,19,22,24,25,37]. Consistent with these views and a recent study [50], our data showed that blockade of spinal 5-HT_3_ receptor function by intrathecal Y25130, a selective 5-HT_3_ receptor antagonist, attenuated mechanical and thermal hypersensitivity following L5 SNL in rats. Interestingly, some studies reported that intrathecal injection of 5-HT_3_ receptor antagonists such as CGP35348 [51] or ondansetron [52] had no preventive effects on mechanical allodynia and/or thermal hyperalgesia in a rat with L5/6 SNL, which conflicts with the study with the same drug ondansetron in the same SNL model [50] and our results. However, we noticed that there were no expected plastic changes of both 5-HT immunoreactive intensity and 5-HT_3_ receptor innervation in the lumbar spinal dorsal horn at 14 d after L5/L6 SNL in the study reported by Peters and colleagues [52]. In contrast, we found a robust increase of tissue Tph-2 level in the RVM [16] at 14 d and a progressive enhancement of tissue 5-HT_3_ receptor expression in the spinal dorsal horn from 1 d to 28 d after nerve injury when compared with that in the sham group (unpublished observations). We suspect that the discrepancies between our positive findings and those reported by Peters et al. [52] could be attributed to the utilization of different neuropathic pain models and different 5-HT_3_ receptor antagonists as well as the absence of more quantitative measures used for the 5-HT_3_ receptor expression and fewer time points measured after injury in their study. In the present study, our data indicate that the effective dose of intrathecal Y25130 for attenuation of behavioral hypersensitivity following SNL did not alter thermal and mechanical thresholds in the sham animals at 14 d after surgery. Thus, we propose that increased descending 5-HT drive and spinal 5-HT_3_ receptor expression after tissue and nerve injury contribute to the maintenance of central sensitization, including glial hyperactivity and neuronal hyperexcitability at the spinal level underlying the development of persistent pain.

We have determined that a number of chemical mediators contribute to the spinal 5-HT_3_ receptor-induced novel spinal signaling cascade that includes the chemokine, fractalkine released from 5-HT_3_ receptor-containing neurons, cytokine IL-18 released from microglia, IL-1β released mainly from astrocytes, enhanced phosphorylation of spinal NMDA receptors, and ultimately behavioral hyperalgesia. Moreover, the mechanisms by which these events are sequentially activated through multiple signaling cascades to link neuron-microglia-astrocyte-neuronal interactions (Figure 8D) is unexpected and novel, and highlights how cellular circuitry and molecular signaling interact in the spinal dorsal horn response to 5-HT_3_ receptor activation. The findings indicate that spinal hyperexcitability or central sensitization underlying the development of hyperalgesia not only depends on the initiation of nociceptive input from primary afferent neurons after tissue and nerve injury [35-37], but also requires the maintenance of descending facilitation from the RVM 5-HT-spinal 5-HT_3_ receptor systems [25,37]. Our study supports the growing evidence that spinal 5-HT_3_ receptors play a crucial role in the cellular and molecular mechanisms of the development and maintenance of persistent pain states.

Our results demonstrate that there are at least three active signaling cascades, including fractalkine and its receptor, CX3CR1, for mediating spinal neuron-to-microglia signaling; IL-18 and its receptor for microglia-to astrocyte signaling; and IL-1β and its receptor for astrocyte-to-neuron signaling, as important components involved in the functional intercellular transduction in the dorsal horn after 5-HT_3_ receptor activation. These findings do not rule out the role of other chemical mediators released from the same neurons, or different subpopulations of neurons (excitatory or inhibitory neurons) or glial cells in the regulation of spinal nociceptive processes. It has been reported that some central terminals of primary afferent neurons express 5-HT_3_ receptors [20,21]. In a recent study, we also showed that 5-HT_3_ receptors in the central terminals of primary afferent neurons are involved in enhanced primary nociceptive afferent activity and excitatory signaling input by increasing TRPV1 function during the maintenance of neuropathic pain [25]. Intrathecal injection of 5-HT_3_ receptor agonists may excite these central terminals to release fractalkine, glutamate and ATP, and directly activate glial cells and even directly enhance NMDA receptor function in dorsal horn neurons. Although these findings suggest other signaling cascades, the converging data in the present study suggest that spinal neuron-glia-neuronal interaction may be particularly important in the 5-HT_3_ receptor-mediated central sensitization associated with intra-RVM 5-HT-dependent descending pain facilitation. Thus, up-regulation of 5-HT_3_ receptor expression in the spinal dorsal horn, following enhanced descending 5-HT drive after nerve injury, may play an important role in glial hyperactivity involved in the maintenance of persistent pain.

Although spinal glial hyperactivity has been reported in acute and persistent pain models [26,29,30,53,54], few studies have investigated the involvement of spinal 5-HT_3_ receptors in spinal glial hyperactivity. In the present study, intrathecal injection of the selective activation of spinal 5-HT_3_ receptors by intrathecal injection of the receptor agonist induced significant up-regulation of GFAP and Iba1. Although Western blot analysis did not show up-regulation of CD11b in the dorsal horn after single i.t. injection of SR57227, immunostaining for CD11b exhibited hypertrophic status of spinal microglia after SR57227, similar to Iba1 labeling. Interestingly, up-regulation of CD11b expression in spinal dorsal horn tissue was observed at 1d after hindpaw inflammation but not 2 h after intrathecal injection of SR57227, suggesting that increase of CD11b expression may require longer-lasting excitatory or nociceptive input on microglia. Molecular depletion of the descending 5-HT system significantly attenuated peripheral inflammation-produced glial hyperactivity in the spinal dorsal horn. These data provide the first evidence that either exogenous or endogenous activation of the 5-HT_3_ receptor results in spinal glial hyperactivity. Moreover, we were interested in the mechanisms by which quiescent spinal glia alter their function in response to 5-HT_3_ receptor activation. Recent studies have demonstrated special expression patterns for chemokines, cytokines and their receptors in spinal cord cells. For example, fractalkine exists in spinal neurons [39,40] and its receptor CX3CR1 is selectively expressed in microglia [40,55]. IL-18 and its receptor are present in spinal microglia and astrocytes, respectively [43]. Consistent with our previous study on the RVM [34,56], we found that IL-1β is mainly expressed in astrocytes but not microglia in the spinal dorsal horn. Its receptor IL-1RI is present in dorsal horn neurons expressing GluNRs. These proteins have been demonstrated to play a role in spinal nociceptive modulation and the development of persistent pain after injury [26,27,29,30]. However, previous studies have not shown a relationship between these proteins and 5-HT_3_ receptor activation in the spinal cord. In addition, extending our recent findings [16,35,56], we showed the colocalization of IL-1RI with the GluNR subunit GluN1R in dorsal horn neurons and with IL-1RI-mediated facilitation of GluN1R phosphorylation after 5-HT_3_ receptor activation. Thus, the IL-1β-mediated amplified signaling from spinal astrocytes further enhances neuronal excitability through signaling coupling with GluNRs in the spinal cord, which plays an important role in neuronal hypersensitivity. Our findings also suggest that activation of spinal 5-HT_3_ receptor is sufficient to induce glial hyperactivity and cytokine release which are necessary for neuronal and behavioral hypersensitivity after 5-HT_3_ receptor activation. The activated glia-mediated positive signaling amplification then sensitizes spinal nociceptive neurons, leading to further neuronal activation and behavioral hyperalgesia. These findings offer new insights into the cellular and molecular mechanisms in the spinal level responsible for descending pain facilitation during the development of persistent pain after tissue and nerve injury.

In the present study, we directly activated the spinal 5-HT_3_ receptor to mimic 5-HT release through descending pain facilitation pathways [16]. We found that intrathecal injection of 10 pmol of the 5-HT_3_ receptor agonist SR 57227 produced thermal and mechanical hypersensitivity that lasted for 4 hours. This observation provides direct evidence that the spinal 5-HT_3_ receptor plays a role in pain facilitation. Activation of 5-HT_3_ receptors in the spinal cord by 5-HT is mediated by the descending excitatory drive from the RVM to the spinal cord [5,24,37]. Consistent with studies with another 5-HT_3_ receptor agonist 2-Me-5H [57,58], we found that intrathecal injection of higher doses of SR 57227 (10 nmol) induced transient analgesia. The different doses used in our experiments may reflect different mechanisms that depend on specific cellular circuits or the particular proteins involved. It has been shown that 5-HT_3_ receptors are predominantly localized in terminals of excitatory axons in the rat superficial dorsal horn and some of these originate from dorsal horn neurons [20,21,59,60]. Although cell bodies expressing these receptors in the dorsal horn are further identified as excitatory neurons [59,61], some 5-HT_3_ receptor-labeled neurons in rat dorsal horn express glutamate decarboxylase (GAD), a marker for GABAnergic neurons [21]. Recent studies have demonstrated in the mouse that some dorsal horn neurons sensitive to 5-HT_3_ receptor agonists were GAD positive [62] and that some 5-HT_3_ receptor mRNA-containing dorsal horn neurons were GAD positive [63]. Thus, 5-HT_3_ receptors appear to be expressed in both excitatory and inhibitory intrinsic neurons and terminals in the spinal dorsal horn. Synaptic plasticity of 5-HT_3_ receptor expression and function in the spinal dorsal horn neurons and the terminals of primary afferent fibers during the development of persistent pain will require further study.

## Conclusions

Our findings demonstrate that activation of neuronal 5-HT3 receptors in the dorsal horn evokes a novel spinal signaling cascade including the chemokine, fractalkine released from 5-HT3 receptor-containing neurons, cytokine IL-18 released from microglia, IL-1β released mainly from astrocytes, and enhanced phosphorylation of NMDA receptors in spinal neurons. This neuronal-glial-cytokine-neuronal signaling cascade may be involved in the mechanisms underlying spinal 5-HT3 receptor-mediated 5-HT-dependent descending facilitation and behavioral hyperalgesia after tissue and nerve injury. These results further support the growing evidence that spinal 5-HT3 receptors play a crucial role in the cellular and molecular mechanisms in the development and maintenance of persistent pain states.

## Methods

### Animals

Adult male Sprague Dawley rats weighing 200–300 g (Harlan, Indianapolis, IN) were used in all experiments. Rats were on a 12 h light/dark cycle and received food and water *ad libitum*. The experiments were approved by the Institutional Animal Care and Use Committee of the University of Maryland Dental School.

### Intrathecal injection

A lumber puncture procedure was adapted according to Hylden and Wilcox [64]. Briefly, rats were anesthetized with 2–3% isoflurane in a gas mixture of 30% O_2_ balanced with nitrogen and placed in a prone position on a styrofoam board with the forelimbs extended rostrally and the hind limbs hanging off the board. A portion of the caudal half of the rat’s back was shaved and scrubbed with providone-iodine solution. A disposable 25-gauge 1-inch needle connected to a 25-μl Luer tip Hamilton syringe was inserted slowly at the intervertebral space between the L4-L5 vertebra and the needle was allowed to penetrate the dura. A quick flick of the tail or a limb indicated entrance into the intrathecal space. Rats awoke within minutes upon the completion of intrathecal injection and termination of anesthesia.

### Intra-RVM microinjection and gene transfer

For intra-RVM microinjection, under anesthesia with 3% isoflurane rats were placed in a Kopf stereotaxic instrument (Kopf Instruments). A midline incision was made after infiltration of lidocaine (2%) into the skin. A midline opening was made in the skull with a dental drill to insert a microinjection needle into the target site. The RVM is termed for collective structures that consist of the midline nucleus raphe magnus (NRM) and the adjacent gigantocellular reticular nucleus α part (NGCα). The coordinates for the NRM were as follows: 10.5 mm caudal to bregma, midline, and 9.0 mm ventral to the surface of the cerebellum [65]. To avoid penetration of the transverse sinus, the incisor bar was set at 4.7 mm below the horizontal plane passing through the interaural line. Animals were subsequently maintained at 1% halothane. For gene transfer, as previously described (Wei et al., [16]), microinjections of the plasmids were performed by delivering Suresilencing™ shRNA plasmid (TCAACATGCTCCATATTGAAT, 0.5 μg/0.5 μl; SuperArray, Frederick, MD, USA) slowly over a 10 min period using a 0.5 μl Hamilton syringe with a 32 gauge needle. The control group underwent identical procedures with injection of the same volume of scrambled shRNA plasmid (ggaatctcattcgatgcatac). Focal electroporation around the RVM area was delivered by seven square wave electric pulses (50 ms, 40 V, 1 Hz; model 2100; A-M Systems, Carlsborg, WA, USA). The wound was closed and the wound margins were covered with a local anesthetic ointment (Nupercainal; Rugby Laboratories), The animals returned to their cages after they recovered from anesthesia.

### Pain models and behavioral testing

To establish a persistent pain model with L5 spinal nerve ligation (L5 SNL), rats were anesthetized with 2–3% isoflurane in a gas mixture of 30% O2 balanced with nitrogen, the left L5 spinal nerve was exposed and tightly ligated with 4–0 soft silk thread. Sham surgery was used as a control. To examine whether there were effects of descending 5-HT depletion on spinal glial hyperactivity induced by peripheral inflammation, complete Freund’s adjuvant (CFA, 50 μl, 25 μg *Mycobacterium tuberculosis*) was injected subcutaneously into the plantar surface of the left hindpaw at 3 d following gene transfer.

Animals were placed in clear plastic chambers on an elevated table and allowed to acclimate for approximately 30 min. Nociceptive responses to thermal and mechanical stimuli were measured. Thermal hyperalgesia was assessed by measuring the latency of paw withdrawal in response to a radiant heat source. A radiant heat stimulus was applied from underneath the glass floor with a high-intensity projector lamp bulb (8 V, 50 W; Osram, Berlin, Germany). The heat stimulus was focused on the plantar surface of each hindpaw, and the paw withdrawal latency (PWL) was determined by an electronic clock circuit. The bulb voltage was adjusted to derive a baseline withdrawal latency (10–12 s) in naive animals. A 20-s cutoff was used to prevent tissue damage. The PWL was tested for three trials with 5-min intervals between each trial. The average of the three trials was then determined. The mechanical sensitivity was measured with a series of calibrated von Frey filaments before and after gene transfer and tissue or nerve injury. An EF_50_ value was defined as the von Frey filament force (g) that produced a 50% frequency of the paw withdrawal responses and was used as a measure of mechanical sensitivity. Body weight and hindpaw diameters were determined before and after gene transfer as well as at 1 and 3 d after inflammation. All behavioral tests were conducted under blind conditions.

### Intra-RVM electrical stimulation

Rats were anesthetized with 1.5% isoflurane and mounted in a stereotaxic apparatus. The stimulation site in the RVM was located stereotaxically as described above. A concentric bipolar stimulating electrode was introduced into the RVM. Trains (2 min on and 30 s off) of stimuli of 0.5 ms square wave pulse were applied with low (10 μA) or high (100 μA) intensity at 10 Hz for 15 min. The sham group received an electrode placement without stimulation. At 30 min after stimulation, sham and treated rats were anesthetized with 2% halothane and decapitated. The spinal dorsal horn tissues at L4-5 were removed for Western blot to examine the expression of CD11b and GFAP.

### Immunohistochemistry

1 h, 2 h and 4 h after intrathecal injection of drugs, rats were deeply anesthetized with pentobarbital sodium (100 mg/kg, i.p.) and transcardially perfused with 200 ml normal saline followed by 500 ml 0.1 M phosphate buffer containing 4% paraformaldehyde (pH = 7.4). The lumber spinal cord was removed, post fixed, and transferred to 20% sucrose overnight. Transverse sections (free-floating, 20 to 40-μm) were cut with a cryostat. The free-floating sections were incubated with relevant antibodies with 1% normal goat sera and 0.3% Triton x-100 overnight at 4°C. After washes in PBS, the sections were incubated with relevant IgGs conjugated to Cy3 or Cy2 (1:500; Jackson ImmunoResearch, West Grove, PA) for 4 h at room temperature or overnight at 4°C. For the double immunofluorescent staining for IL-18 and NeuN, GFAP or Iba1, the tyramide signal amplification (PerkinElmer Life Sciences, Boston, MA) fluorescence procedures [66] were used to detect staining for goat anti-IL-18 polyclonal antibody (1:10000; R & D Systems). Following washes, the stained sections were mounted on gelatin-coated slides and coverslipped with Vectashield (Vector Laboratories). Slides were examined with a Nikon fluorescence microscope and images were captured with a CCD Spot camera. A Bio-Rad laser scanning confocal microscope was also used for higher magnification and colocalization.

### Western blot

Rats were sacrificed 1 h, 2 h and 4 h after intrathecal injection of drugs. The L5-6 spinal cord was rapidly removed and the dorsal half was separated and frozen on dry ice. The tissues were homogenized in solubilization buffer (50 mM Tris HCl, pH 8.0, 150 mM NaCl, 1 mM EDTA, 1% NP-40, 0.5% deoxycholic acid, 0.1% SDS, 1 mM Na_3_VO_4_, 1 U/ml aprotinin, 20 μg/ml leupetin, 20 μg/ml pepstatin A). The homogenate was centrifuged at 14,000 rpm for 10 min at 4°C. The supernatant was removed. The protein concentration was determined using a detergent-compatible protein assay with a bovine serum albumin standard. Each sample contains proteins from one animal. Protein samples (35 μg) were separated on 7.5% SDS-PAGE and blotted on a nitrocellulose membrane (GE Healthcare, Piscataway, NJ). The blots were blocked with 5% milk in Tris-buffered saline (TBS) for 30 min and then incubated with respective antibodies overnight at 4°C. The membrane was washed with TBS and incubated with anti-goat/mouse/rabbit IgG (1:1000; Santa Cruz Biotechnology, Santa Cruz, CA) for 1.5 h at room temperature. The immunoreactivity was detected using enhanced chemiluminescence (ECL; GE Healthcare). Some blots were further stripped in a stripping buffer (Thermo Scientific) for 30 min at 50°C. The loading and blotting of equal amount of protein were verified by reprobing the membrane with anti-β-actin antiserum (Sigma). Specific expression band for the targeted proteins was identified with the marker bands for the expected molecular weight (KDa).

### Data analysis

Data were presented as means ± SEM, and analyzed using one- or two.-way ANOVA. The significant differences between the groups were determined by a post-hoc test. P < 0.05 is considered significant for all cases. For Western blot analysis, the ECL-exposed films were digitized and immunoreactive bands were quantified by U-SCAN-IT gel (version 4.3; Silk Scientific, Orem, UT). The relative protein levels were obtained by comparing the respective specific band to the β-actin control from the same membrane. The deduced ratios were further normalized to that of the naive rats on the same membrane and illustrated as the percentage of the naive controls. Raw data (ratios of the respective band over β-actin) were used for statistical comparisons.

### Drugs and antibodies

The following drugs were used for intrathecal injection: 5-HT_3_ receptor agonist SR-57227 hydrochloride (TOCRIS, Ellisvlle, MO), 5-HT_3_ receptor antagonist Y-25130 (TOCRIS, Ellisvlle, MO), fractalkine (aa 22–100, R & D Systems), neutralizing antibody against rat CX3CR1 (CX3CR1 Ab, Torrey Pines Biolabs, Houston, TX) and IL-1β receptor (IL-1ra, Amgen, Thousand Oaks, CA), anti-IL-18 receptor (IL-18R Ab, R & D Systems), recombinant rat IL-18 (R&D systems) and IL-1β (PeproTech).

The following antibodies were used for western blot and immunohistochemistry: The polyclonal primary antibodies were used in the following dilutions: anti-5-HT_3_ receptor (1:500, Calbiochem, Gibbstown, NJ), anti-glial fibrillary acidic proteins (GFAP,1:10000, Millipore, Bedford, MA; or 1:1000, Chemicon, Temecula, CA), anti-S100β (1:1000, Millipore, Bedford, MA), anti-Iba1 (1:1000; Wako, Osaka, Japan), anti-fractalkine (1:1000) (Novus Biological, Littleton, CO), anti-CX3CR1 (1:1000, Torrey Pines Biolabs, Houston, TX), anti-IL-18 (1:400, R & D Systems), anti-IL-18R (1:500, R & D Systems), anti-IL-1β (1:500, Endogen, Rockford, IL), anti-IL-1R (1:500, Santa Cruz Biotechnology), and anti-p-GluN1 (or NR1) ser896 (1:1000, Millipore, Bedford, MA). The monoclonal primary antibodies were used in the following dilutions: anti-CD11b (clone OX-42, 1:1000, Serotec, Raleigh, NC), anti-NeuN, (1:1000, Millipore, Bedford, MA; or 1:2000, Chemicon), anti-GluN1 (1:1000, Millipore) and anti-β-actin (Sigma-Aldrich).

## Competing interests

The authors declare that they have no competing interest.

## Authors’ contribution

WG contributed to western blotting analysis and behavioral tests, analyzed and interpreted data; KM contributed to IHC studies; RD designed research, participated in interpretation and revised the manuscript. MG contributed to IHC, western blotting and behavioral experiments, analyzed and interpreted data; ML carried out western blotting and behavioral experiments; JL carried out IHC and behavioral experiments; SZ carried out behavioral tests; KR designed research and interpreted data; KN participated in data analysis and interpretation; FW contributed to the conception and design of the studies, analysis and interpretation of data, writing and revising the manuscript. All authors read and approved the final manuscript.
